# Clinical characteristics and long-term management of prepubertal testicular teratomas: a retrospective, multicenter study

**DOI:** 10.1007/s00431-023-04859-8

**Published:** 2023-02-16

**Authors:** Guanglun Zhou, Fenglan Sun, Xin Yu, Ruifeng Huang, Xiaodong Liu, Yaoling Ouyang, Zhilin Yang, Shoulin Li

**Affiliations:** 1 Department of Urology and Laboratory of Pelvic Floor Muscle Function, Shenzhen Children’s Hospital, Futian District, Shenzhen, 518000 Guangdong China; 2 Department of Laboratory Medicine, Jingzhou Hospital Affiliated to Yangtze University, Jingzhou, 434020 China; 3 Department of Urology, Anhui Provincial Children’s Hospital, Hefei, China; 4 Department of Gastroenterology and Zhengzhou Key Laboratory of Children’s Digestive Diseases, Children’s Hospital Affiliated to Zhengzhou University, Henan Children’s Hospital, Zhengzhou Children’s Hospital, Zhengzhou, Henan China

**Keywords:** Testis, Teratoma, Testis-sparing surgery, Treatment

## Abstract

Prepubertal testicular teratomas are rare tumors with limited practical guidance for their management. This study aimed to analyze a large multicenter database to establish the optimal management of testicular teratomas. We retrospectively collected data on testicular teratomas in children younger than 12 years who underwent surgery without postoperative chemotherapy in three large professional children’s institutions in China between 2007 and 2021. The biological behavior and long-term outcomes of testicular teratomas were analyzed. In total, 487 children (with 393 mature teratomas and 94 immature teratomas) were included. Among mature teratomas, 375 cases were testis-sparing, 18 were orchiectomies, 346 were operated through the scrotal approach, and 47 underwent the inguinal approach. The median follow-up period was 70 months, and no recurrence or testicular atrophy was observed. Among the children with immature teratomas, 54 underwent testis-sparing surgery, 40 underwent orchiectomy, 43 were operated through the scrotal approach, and 51 were operated through the inguinal approach. Two cases of immature teratomas with cryptorchidism had local recurrence or metastasis within 1 year of the operation. The median follow-up duration was 76 months. No other patients had recurrence, metastasis, or testicular atrophy.

*  Conclusion*: Testicular-sparing surgery is the first treatment choice for prepubertal testicular teratomas, with the scrotal approach being a safe and well-tolerated strategy for these diseases. Additionally, patients with immature teratomas and cryptorchidism may have tumor recurrence or metastasis after surgery. Therefore, these patients should be closely followed up in the first year after surgery.

**Table:** 

**What is Known:** *• There is a fundamental difference between testicular tumours in childhood and those in adulthood - not only in terms of the difference and incidence but also in terms of histology.* *• For surgical techniques, the inguinal approach is recommended for the treatment of testicular teratomas in children.*	
**What is New:** *• The scrotal approach being a safe and well-tolerated strategy for testicular teratomas in children.* *• Patients with immature teratomas and cryptorchidism may have tumor recurrence or metastasis after surgery. These patients should be closely followed up in the first year after surgery.*

**What is Known:**

*• There is a fundamental difference between testicular tumours in childhood and those in adulthood - not only in terms of the difference and incidence but also in terms of histology.*

*• For surgical techniques, the inguinal approach is recommended for the treatment of testicular teratomas in children.*

**What is New:**

*• The scrotal approach being a safe and well-tolerated strategy for testicular teratomas in children.*

*• Patients with immature teratomas and cryptorchidism may have tumor recurrence or metastasis after surgery. These patients should be closely followed up in the first year after surgery.*

## Introduction

Testicular germ cell tumors are uncommon in children, with an incidence of approximately 0.5–2.0/100,000 individuals [1]. Further, there are fundamental histological differences between testicular tumors in children and adults [2]. Notably, most testicular tumors in prepubertal boys are benign [2].

Teratomas are the most common testicular tumors in children [3]. Some small-sample case studies have reported the diagnosis and management of teratomas [4–7]. However, the following questions still need to be addressed: (a) whether adjuvant chemotherapy is needed for immature teratomas after surgery, (b) the long-term impact of testis-sparing surgery for immature teratomas, (c) decision-making factors for a testis-sparing approach for surgery, and (d) whether different surgical approaches affect prognosis.

Therefore, in this study, we analyzed a large multicenter database to explore the biological behavior of testicular teratomas and assess their long-term outcomes after surgery. In addition, we aimed to identify factors affecting the prognosis of testicular teratomas.

## Methods

### Study design and population

This retrospective multicenter study was approved by the Institutional Review Committees of Henan Children’s Hospital, Anhui Children’s Hospital, and Shenzhen Children’s Hospital. The parents or guardians of all participants provided written informed consent before treatment.

This study collected data from three professional children’s institutions, as mentioned above, between January 2007 and December 2021. The inclusion criteria were as follows: (1) children with testicular tumors who underwent surgery and were diagnosed with testicular teratomas by pathology after surgery, (2) younger than 12 years old, and (3) follow-up time after surgery was ≥ 6 months. Exclusion criteria included: (1) non-primary testicular teratomas; (2) mixed testicular teratomas (yolk sac tumor and teratomas); (3) yolk sac tumors; and (4) cases with missing data. Ten patients were excluded due to missing data, including four patients without tumor size data, four without preoperative ultrasonography results, and two without postoperative pathological results. These children received surgical treatment and were followed up as planned. All patients underwent ultrasonographic examination of the reproductive system before surgery. Abdominal ultrasonography before surgery was performed on 375 children with mature teratomas and 89 children with immature teratomas.

The collected clinical data included age at surgery, clinical manifestations, concomitant diseases, ultrasonography results, preoperative serum alpha-fetoprotein (AFP) levels, treatment details, tumor size, histopathology, and long-term outcomes. Age at surgery, AFP levels, and tumor size were included as continuous variables with normal distribution, and other variables were included as categorical variables. Tumor size was defined as the maximum diameter of the tumor measured intraoperatively. Immature teratomas were classified into grades 1–3 following the Norris pathological classification [8].

If the testis was located in the scrotum, children with benign tumors were classified according to their preoperative AFP and ultrasonography results. The choice between the inguinal or scrotal approaches was made based on the surgeon’s judgment. If testicular tumors were associated with cryptorchidism, the inguinal or laparoscopic approach was performed according to the position of the testis and the surgeon’s clinical experience. During the operation, if the tumor boundary was clear and normal testicular tissue was still present after the tumor had been completely removed, testis-sparing surgery was used when intraoperative frozen sections confirmed the teratomas. Testicular resection was performed if the tumor could not be completely removed during the operation, no normal testicular tissue was found, or frozen section pathology indicates malignant results.

### Follow-up

Patients were followed up every 3 months in the first year after surgery, with each follow-up including a physical examination, AFP determination, and ultrasonography; then, patients were followed up once a year.

### Statistical analysis

Continuous variables are presented as mean ± standard deviation; categorical variables are presented as frequencies and percentages (%). The chi-square or Fisher’s exact test was used to assess categorical variables, and the Student’s *t*-test for continuous variables. The normality of variables was assessed using the Kolmogorov–Smirnov test. Statistical significance was set at *P* < 0.05. Data analysis was performed using SPSS version 23.0 (IBM, New York, NY, USA).

## Results

### Overall results

This study included 487 children: 482 patients with unilateral testicular tumors (226 cases on the right and 256 on the left), five with bilateral testicular tumors. The median age at surgery was 17 months (range: 29 days–123 months). In this study, 446 patients (91.6%) presented with a scrotal mass, 24 had scrotum emptiness, and 17 had other symptoms. A hydrocele was found in 30 patients, cryptorchidism in 26, and inguinal hernia in 13. The median follow-up duration was 71 months (range: 7–161 months).

Findings based on the intraoperative frozen sections were consistent with the postoperative pathological results in all children. Postoperative pathology revealed 393 and 94 cases of mature and immature teratomas, respectively. Patients with mature teratomas were younger and had higher AFP levels and had larger testicular tumor diameters than did those with mature teratomas (*P* < 0.05). The demographic characteristics, tumor characteristics, and outcomes of all patients are summarized in Table 1.

**Table 1:** Clinical characteristics of testicular teratomas in children

Characteristics	Mature teratomas	Immature teratomas	*P* value
*N*	393	94	
Age at surgery (months)	19.3 ± 4.8	9.2 ± 2.7	0.001
AFP level (ng/ml)	20.5 ± 6.2	75.3 ± 19.5	0.001
Tumor size (cm)	1.9 ± 0.5	2.9 ± 0.6	0.001
Surgery methods			0.001
Testis-sparing	375 (95.4%)	54 (57.4%)	
Orchiectomy	18 (4.6%)	40 (42.6%)	
Outcomes	0 (0%)	2 (2.1%)	0.083
Metastasis	0 (0%)	1 (1.0%)	
Recurrence	0 (0%)	1 (1.0%)	

### Mature teratomas

Among patients with mature teratomas, the median AFP level of patients > 12 months old was within the normal range: while that of patients < 12 months old was 2.2 ng/ml. The median operative age and tumor size of the mature teratomas were 19 months and 1.9 cm, respectively. Cryptorchidism was observed in 16 patients. Ultrasonography revealed that 348 cases had sufficient testicular parenchyma, 45 cases had no obvious or described testicular parenchyma, 329 tumors had clear boundaries, and 64 tumors had unclear or undescribed boundaries. A total of 346 cases were operated via the scrotum and 47 via the inguinal approach. Three cases were assisted by laparoscopy. There were 375 cases with testis-sparing surgery, 356 cases undergoing intraoperative rapid frozen section, and 18 with orchiectomy. The tumor size ranged from 1.3 to 4.0 cm. The median follow-up duration was 70 months (range: 7–140 months), with no recurrence observed in any patient. None of the patients who underwent testicular-sparing surgery had testicular atrophy.

### Immature teratomas

Among patients with immature teratomas, the median AFP level of patients > 12 months old is within the normal range: while that of patients < 12 months old was 82.2 ng/ml. The median operative age and tumor size of the immature teratomas were 9 months and 2.8 cm, respectively. Cryptorchidism was observed in ten patients. Ultrasonography revealed that 44 cases had sufficient testicular parenchyma, 50 had no obvious or described testicular parenchyma, 43 tumors had clear boundaries, and 51 tumors had unclear or undescribed boundaries. A total of 43 patients underwent surgery via the scrotum and 51 via the inguinal approach. Three cases were assisted by laparoscopy. There were 54 with testis-sparing surgery, 52 cases undergoing intraoperative rapid frozen section, and 40 with orchiectomy. The tumor size ranged from 1.3 to 3.8 cm. According to the Norris pathological classification, postoperative pathological results showed 19 cases of grade 1, 32 cases of grade 2, and 43 cases of grade 3. The median follow-up was 76 months (range: 16–161 months), with only two cases exhibiting local recurrence or metastasis. None of the patients who underwent testicular-sparing surgery had testicular atrophy.

One patient had retroperitoneal lymph node metastasis 6 months after an orchiectomy. The patient had a surgical age of 9 months, a preoperative AFP of 77.0 ng/ml, a tumor size of approximately 4.9 cm, and no normal testicular tissue during the operation. The patient underwent a retroperitoneal lymphadenectomy and chemotherapy (including cisplatin, etoposide, and bleomycin). The pathology of the metastasis was similar to that of primary immature testicular teratomas. No recurrence or metastasis was observed during the 4-year follow-up. Another patient underwent testis-sparing surgery, and local recurrence of the testicular tumor was observed 8 months after the operation. The patient had an age of 8 months, a preoperative AFP of 64.6 ng/ml, and normal testicular tissue after complete surgical resection of the tumor. Subsequently, the patient underwent testis-sparing surgery, complete resection of the tumor was performed during the operation, and the pathology of the recurrent testicular tumor was similar to that of the primary immature teratoma. No recurrence or metastasis was observed during the 3-year follow-up. Interestingly, both patients had cryptorchidism before surgery.

## Discussion

Prepubertal testicular teratomas are rare tumors, and practical clinical guidance for these tumors is limited [9]. To establish optimal management of testicular teratomas, we undertook multicenter coordination to collect a large data series.

Testicular-sparing surgery should be considered for children with testicular teratomas to avoid removing the testes if preservation is possible. Testicular preservation should be prioritized in children because it benefits children’s psychological and physical development and maintains fertility and endocrine functions [10]. Measurement of AFP levels and ultrasonography are important diagnostic tools for testicular tumors before surgery [9]. In our clinical practice, normal AFP levels and teratomas diagnosed by ultrasonography can be used as two reference for the preoperative evaluation of benign testicular tumors. Previous studies showed that tumor size is an important predictor of testicular preservation or removal [10–12]. In 1999, Heidenreich and Hofmann reported that testicular-sparing surgery should not be performed on tumors larger than 2.0 cm in diameter [11]. In 2003, Steiner et al. noted that testicular-sparing surgery could be considered when the diameter of tumors is less than 2.5 cm [10]. In recent years, Li et al. claimed that testicular-sparing surgery should be performed if the diameter of the tumor is less than 3.0 cm [12]. However, Patel et al. considered that the number of normal parenchyma was underestimated due to the compression and thinning of the testicular parenchyma caused by large tumors; they suggested that surgeons should perform testicular-sparing surgery, even if there are large and benign tumors and seemingly small normal parenchyma [13]. In this study, testicular tumor size was not considered a necessary indicator for testicular preservation. During the operation, we tried to completely remove testicular tumors and retain normal testicular tissue to the maximum extent. Our data show that even when the diameter of the testicular tumor exceeds 3 or 4 cm, the tumor can be completely removed, and normal testicular parenchyma can still be preserved. Fortunately, these patients showed no evidence of testicular atrophy after a long follow-up. With the development of surgical techniques, especially microscopic techniques, more testicles may be preserved in children with large testicular teratomas.

Radford et al. reported that frozen sections provide a basis for testis-sparing surgery [9]. In this study, there was a high degree of consistency between the results from frozen sections and the final histopathological examination. Our data showed that examining an intraoperative frozen section discriminates between malignant and benign testicular tumors, allowing testis-sparing surgery to be performed safely and reliably. A previous study found that 62.1% (23/37) of doctors performed testicle-sparing surgery for testicular tumors; however, there was no diagnosis of intraoperative frozen sections during surgery [9]. In contrast, 29.7% (11/37) of doctors performed a second operation based on the final histopathological examination to complete the treatment [9]. Examining frozen sections may help avoid reoperations in these patients. Therefore, frozen sections are necessary for testicular-sparing surgery.

Few studies reported the recurrence or metastasis of testicular teratomas after orchiectomy or testicular-sparing surgery. Hisamatsu et al. reported one mature teratoma recurrence after testicular-sparing surgery [4]. This study showed that testicular-sparing surgery was performed during the operation of many mature teratomas, and no recurrence was observed during a long-term follow-up. Thus, complete resection of mature teratomas during surgery is critical to avoid recurrence. Unlike mature teratomas, immature teratomas have incompletely differentiated tissue components, which may cause some uncertainty in their biological behavior [14]. In this study, testicular-sparing surgery was performed in most cases, and no chemotherapy was administered after surgery in all cases. Two children with immature teratomas and cryptorchidism had tumor recurrence and metastasis, but no similar phenomenon was found in immature teratomas with the normal testicular position. Whether this phenomenon is related to the abnormal location of immature teratomas requires further investigation. Similarly, Hasegawa et al. reported that a 3-month-old boy with immature teratoma and cryptorchidism developed retroperitoneal lymph node metastasis 3 months after orchiectomy, suggesting that postoperative chemotherapy combined with orchiectomy may help treat prepubertal testicular immature teratomas with cryptorchidism [15]. However, there are different interpretations of the observation. Although adjuvant chemotherapy could be considered for these patients, in this study, none of the patients with recurrence or metastasis of immature teratomas had the disease after salvage therapy. Despite this, no recurrence or metastasis was found in the other eight children with immature teratomas with cryptorchidism during long-term follow-up. In addition, patients with immature teratomas are usually younger, and the potential side effects of chemotherapy should be fully considered. Therefore, we recommend closely monitoring patients with immature teratomas and cryptorchidism in the first year after surgery, and a regular follow-up should be carried out annually after that. Additionally, retroperitoneal ultrasonography examination should be included in the follow-up program.

The inguinal approach is currently the main method to treat testicular tumors in children [9]. Its benefits include good surgical vision and the prevention of the spread of testicular tumor cells through blood vessel clamping [9]. In this study, most patients underwent the scrotal approach, which can also fully expose the spermatic cord vessels after spermatic cord dissociation and ensure blockage of blood flow during surgery. In clinical practice, the scrotal approach does not only satisfactorily achieve testicular-sparing resection or orchiectomy but also reduces surgical trauma. In addition, there is no conclusive evidence in the literature that clamping blood vessels can prevent tumor spread in testicular teratomas [9]. In this study, tumor metastasis or recurrence was not observed in children who underwent the operation of testicular teratomas by the scrotal approach. Therefore, our data support using the scrotal approach as a safe and well-tolerated technique for treating prepubertal testicular teratomas.

AFP detection is helpful in the differential diagnosis of malignant and benign teratomas before surgery and is an important indicator for monitoring metastasis and recurrence after surgery [9]. However, AFP may increase physiologically in patients younger than 12 months, reducing its diagnostic value. Ross et al. reported that although the AFP level of testicular teratomas under 1 year of age is increased physiologically, it is usually below 100 ng/ml [16], which is consistent with our research results.

Our study has some limitations. It is a retrospective study with potential selection bias. The operation was performed by multiple surgeons, and the choice of surgical techniques may affect the results. In addition, the testicular teratomas in this study were not classified according to the new pathology guidelines proposed by the World Health Organization in 2016 [17], reducing the generalizability of the results compared to testicular teratomas classified according to the new pathology guidelines.

## Conclusions

Testicular-sparing surgery is the first choice for prepubertal testicular teratomas, and tumor size should not be a determinative factor for testicular-sparing surgery. Testicular-sparing surgery requires frozen sections to avoid unnecessary reoperation. Compared with people with a normal testicular position, patients with immature teratomas and cryptorchidism are more likely to have tumor recurrence or metastasis after surgery. Therefore, these patients should be followed up closely in the first year after surgery. The scrotal approach is safe and well-tolerated for prepubertal testicular teratomas.

## Data Availability

The datasets used and/or analyzed during the current study are available from the corresponding author on reasonable request.

## Abbreviation

AFP: Alpha-fetoprotein
